# Draft genome sequence of a potential probiotic *Pediococcus acidilactici* RTMP1 isolated from fecal samples of Speckled goats in South Africa

**DOI:** 10.1128/mra.00871-25

**Published:** 2025-10-13

**Authors:** Mamosatjana Rittah Modiba, Goitsemang Makete, Kazeem Adekunle Alayande, Ayanda Mavis Ngxumeshe, Ephodia Sihlangu, Tshifhiwa Paris Mamphogoro

**Affiliations:** 1 Gastro-Intestinal Microbiology and Biotechnology Unit, Agricultural Research Council Animal-Production 623417 , Pretoria, South Africa; 2 Department of Animal Sciences, Tshwane University of Technology 56412, Pretoria, South Africa; 3 Unit for Environmental Sciences and Management, North-West University 56405, Potchefstroom, South Africa; University of Maryland School of Medicine, Baltimore, Maryland, USA

**Keywords:** *Pediococcus acidilactici*, Speckled goats, genome assembly, probiotic activities

## Abstract

Here, we report the draft genome sequence of *Pediococcus acidilactici* RTMP1 isolated from fecal samples of Speckled goats. This bacterium (scaffolds = 54; genome size = 1,973,216 bp; coding sequences = 1,870; N_50_ = 59,077; GC content = 42%) encodes for genes attributed to probiotic characteristics.

## ANNOUNCEMENT

*Pediococcus acidilactici* is a lactic acid bacterium (LAB) that has presence in diverse habitats, including gastrointestinal systems and fermented foods (1). This bacterium has received a lot of interest due to its diversity and prospective uses in biotechnology and food preservation (2). Its resilience to acidic and bile conditions, along with its ability to produce antimicrobial compounds, makes it a promising candidate for probiotic applications in livestock animal health (3, 4). Therefore, studying the genome of this bacteria may help predict the genes responsible for the above-mentioned valuable traits.

We sequenced the genome of *P. acidilactici* strain RTMP1, isolated from goat fecal samples. A total of 20 fresh goat fecal samples were collected from the Small-Stock Division of the Agricultural Research Council, Animal Production Unit in Irene, Gauteng, South Africa (25°53′59.60″S, 28°12′51.60″E). Fecal collection was observed based on the protocol reviewed and approved by the Agricultural Research Council-Animal Production Institute Ethics Committee (APIEC24/04) and Tshwane University of Technology Animal Research Ethics Committee (AREC-010411-009). Bacterial cells were isolated through 10-fold serial dilution of each sample (1 g in 9 mL) in sterile 0.85% saline solution, and thereafter, plated on de Man, Rogosa Sharpe (MRS) agar, incubated anaerobically using an Anaerobic Gas Pack (Oxoid) for a period of 48 h at 37°C. After incubation, discrete colonies were sub-cultured onto MRS agar, before being streaked for pure culture. The pure culture in MRS broth was then stored in 50% (vol/vol) glycerol at −80°C.

**Fig 1:**
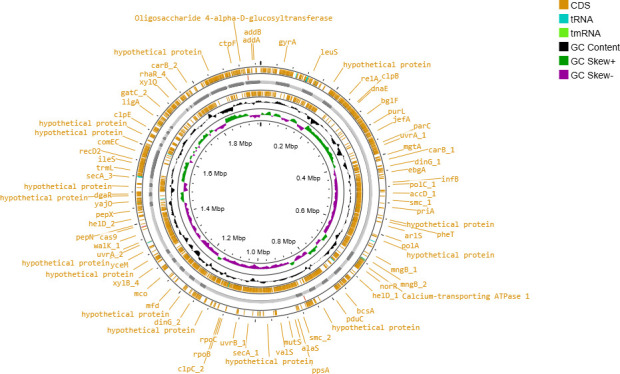
Circular representation of *P. acidilactici* RTMP1 genome, highlighting annotated features, such as gene distribution, CDS, tRNA, tmRNA, and GC Content. Figure created using ProkSee (https://proksee.ca).

The genome of RTMP1 was extracted from overnight culture in MRS broth using a Quick-DNA miniprep kit (Zymo Research, Irvine, CA, USA) following the manufacturer’s instructions. A NanoDrop (ThermoFisher Scientific, Carlsbad, CA, USA) was used to determine the concentration of extracted DNA. A sequencing library was prepared using the NEBNext Ultra II FS DNA library prep kit (New England Biolabs, Ipswich, MA) and sequenced on an Illumina NextSeq 550 platform with standard operating procedures. A total of 9,054,313 paired-end 2 × 150 bp reads generated were analyzed via KBase platform (5). The quality of the reads was evaluated using FastQC v0.11.5 (6), and sequence adaptors and low-quality reads were removed using Trimmomatic v0.36 (7). The genome was assembled *de novo* using SPAdes v3.13.0 (8) yielding 54 scaffolds with a coverage of 1,376.6×. The assembly produced a genome sequence of 1,973,216 bp long, with a G + C content of 42% and an N_50_ value of 59,077. Genome completeness and contamination were performed using CheckM v1.2.2 (9) and were 88.72% and 1.54%, respectively. Gene annotation was performed using the PGAP v6.3 (10) identifying 1,870 protein-coding genes, 50 tRNAs and 54 RNA as well as discovering probiotic genes involved in immunomodulation against pathogens, stress tolerance, acid tolerance, bile salt tolerance, adhesion to epithelial cells, and antioxidant production (Fig. 1 and Table 1), all potentially related to probiotic actions (11, 12). The genome was assigned to *P. acidilactici* via GTDB-Tk v2.3.2 (13) and had the pairwise ANI value of 99.15% average nucleotide identity (ANI) with *P. acidilactici* DSM 20284 (GCF_000146325.1), confirming its affiliation to the *Pediococcus acidilactici* taxonomic group. In each step, default parameters were used except where otherwise noted.

**TABLE 1:** Genes potentially related to probiotic functions of *Pediococcus cidilactici* RTMP1^*a*^

Gene	Function	Locus tag	NCBI protein accession no.	Product
*dlt*D	Immunomodulation	ACSR7A_00875	MGL3993017.1	D-alanyl-lipoteichoic acid biosynthesis protein DltD
*dlt*C		ACSR7A_00880	MGL3993018.1	D-alanine—poly (phosphoribitol) ligase subunit DltC
*dlt*B		ACSR7A_00885	MGL3993019.1	D-alanyl-lipoteichoic acid biosynthesis protein DltB
*dlt*A		ACSR7A_00890	MGL3993020.1	D-alanine—poly(phosphoribitol) ligase subunit DltA
*clp*B	Stress tolerance	ACSR7A_01290	MGL3993100.1	ATP-dependent chaperone clpB
*gro*ES		ACSR7A_06825	MGL3994183.1	Co-chaperone GroES
*hsl*V		ACSR7A_01805	MGL3993202.1	ATP-dependent protease subunit hslV
*hsl*U		ACSR7A_01810	MGL3993203.1	ATP-dependent protease ATPase subunit hslU
*dna*J		ACSR7A_02235	MGL3993287.1	Molecular chaperone dnaJ
*dna*K		ACSR7A_02240	MGL3993288.1	Molecular chaperone dnaK
*hrc*A		ACSR7A_02250	MGL3993290.1	Heat inducible transcriptional repressor hrcA
*clp*P		ACSR7A_06655	MGL3994150.1	ATP-dependent Clp endopeptidase proteolytic subunit ClpP
*clp*X		ACSR7A_07790	MGL3994365.1	ATP-dependent Clp protease ATP-binding subunit ClpX
*pyk*	Acid tolerance	ACSR7A_01310	MGL3993104.1	pyruvate kinase
*atp*D		ACSR7A_04685	MGL3993769.1	F0F1 ATP synthase subunit beta
*atp*A		ACSR7A_04695	MGL3993771.1	F0F1 ATP synthase subunit alpha
*atp*H		ACSR7A_04700	MGL3993772.1	ATP synthase F1 subunit delta
*atp*F		ACSR7A_04705	MGL3993773.1	F0F1 ATP synthase subunit B
*atp*E		ACSR7A_04710	MGL3993774.1	F0F1 ATP synthase subunit C
*atp*B		ACSR7A_04715	MGL3993775.1	F0F1 ATP synthase subunit A
*rpl*I	Bile salt tolerance	ACSR7A_00285	MGL3992920.1	Large ribosomal protein L9
*rps*U		ACSR7A_01225	MGL3993087.1	Small ribosomal protein S21
*rps*A		ACSR7A_01370	MGL3993116.1	small ribosomal protein S1
*rps*B		ACSR7A_02335	MGL3993307.1	Small ribosomal protein S2
*rpl*S		ACSR7A_02465	MGL3993333.1	Large ribosomal protein L19
*rps*P		ACSR7A_02490	MGL3993338.1	Small ribosomal protein S16
*rpm*B		ACSR7A_02545	MGL3993349.1	Large ribosomal protein L28
*rpm*A		ACSR7A_02665	MGL3993373.1	Large ribosomal protein L27
*rpl*U		ACSR7A_02765	MGL3993375.1	Large ribosomal protein L21
*gln*A		ACSR7A_02715	MGL3993382.1	Type I glutamate—ammonia ligase
*rpm*F		ACSR7A_02860	MGL3993411.1	Large ribosomal protein L32
*rpl*T		ACSR7A_02910	MGL3993421.1	Large ribosomal protein L20
*rpm*I		ACSR7A_02915	MGL3993422.1	Large ribosomal protein L35
*rps*D		ACSR7A_04580	MGL3993749.1	Small ribosomal protein S4
GNAT		ACSR7A_06575	MGL3994134.1	GNAT family N-acetyltransferase
LPXTG	Adhesion	ACSR7A_01735	MGL3993188.1	LPXTG cell wall anchor domain-containing protein
*lsp*A		ACSR7A_02035	MGL3993248.1	Signal peptidase II
*tpi*A		ACSR7A_06625	MGL3994144.1	Triose-phosphate isomerase
*cel*B		ACSR7A_07315	MGL3994279.1	PTS cellobiose transporter subunit IIC
*tuf*		ACSR7A_07780	MGL3994363.1	Elongation factor Tu
*trx*A	Antioxidant	ACSR7A_00180	MGL3992899.1	Thioredoxin
*msr*B		ACSR7A_01870	MGL3993215.1	Peptide-methionine (R)-S-oxide reductase MsrB
*msr*A		ACSR7A_01875	MGL3993216.1	Peptide-methionine (S)-S-oxide reductase MsrA
*nrd*H		ACSR7A_05580	MGL3993938.1	Glutaredoxin-like protein NrdH
*trx*B		ACSR7A_06705	MGL3994159.1	Thioredoxin-disulfide reductase

^*a*^ NCBI, National Center for Biotechnolgy Information.

This genome offers a platform for future comprehensive comparative genomic analysis of *Pediococcus* species and understanding of deeper insights into functional annotation and evolutionary relationships.

## Data Availability

This whole-genome shotgun sequencing project has been deposited at DDBJ/ENA/GenBank under the accession number JBPURR000000000. The version described in this paper is the first version. The SRA accession number is SRR34674031, the BioProject accession number is PRJNA1291987, and the BioSample accession number is SAMN49984124.

## AFTER EPUB

*[This article was published on 13 October 2025 with errors in the Acknowledgements and Data Availability statement. These production errors were corrected in the current version, posted on 10 November 2025.]*
